# The Emergence of Animal Management in the Southern Levant

**DOI:** 10.1038/s41598-018-27647-z

**Published:** 2018-06-18

**Authors:** Natalie D. Munro, Guy Bar-Oz, Jacqueline S. Meier, Lidar Sapir-Hen, Mary C. Stiner, Reuven Yeshurun

**Affiliations:** 1 Department of Anthropology, Unit 1176, 354 Mansfield Road, University of Connecticut, Storrs, CT 06250 USA; 2 Zinman Institute of Archaeology, University of Haifa, Mount Carmel, 31905 Haifa Israel; 3 Department of Anthropology, Trent University, DNA Block C, 2140 East Bank Drive, Peterborough, ON K9J 7B8 Canada; 4 Department of Archaeology and Ancient Near Eastern Cultures, Tel Aviv University, Tel Aviv, 6997801 Israel; 5 School of Anthropology, P.O. Box 210030, University of Arizona, Tucson, AZ 85721-0030 USA

## Abstract

Our compilation of zooarchaeological data from a series of important archaeological sites spanning the Epipaleolithic through Pre-Pottery Neolithic B periods in the Mediterranean Hills of the southern Levant contributes to major debates about the beginnings of ungulate management in Southwest Asia. The data support an onset of ungulate management practices by the Early PPNB (10,500–10,000 cal. BP), more than 500 years earlier than previously thought for this region. There is a clear developmental connection between reduced hunting intensity and the uptake of ungulate management, confirming that this process began in response to local, density-dependent demographic factors. The early process of goat domestication in the southern Levant appears to have been overwhelmingly local. This may have been true for cattle and pigs as well. Nevertheless, the loose synchrony of animal management trends across Southwest Asia was undoubtedly enabled by large-scale social networks that transmitted knowledge. The results add to growing evidence that animal management processes followed multiple regional evolutionary pathways within the Fertile Crescent.

## Introduction

Because of its profound impact on all aspects of human sociocultural life, the forager-farmer transition has been subject to intense scrutiny. Its investigation has an especially rich and dynamic history in Southwest Asia, home to the earliest evidence for plant and animal management and domestication^1,2^. Although the amount and quality of data have increased tremendously over the last few decades, the details of domestication processes and associated explanatory models are under continual revision. Current controversies concern the timing, background conditions, and nature of domestication trends. While the Fertile Crescent is widely recognized as a heartland of plant and animal domestication, there is less agreement about the areas over which certain species came under management and human-induced genetic alteration^1,3^. Early indications of the ungulate domestication process include human control over the reproduction or culling of wild type animals, rather than evidence of extensive genetic or morphologic changes. Did management lead to domestic variants in only one place, or were there concurrent evolutionary hotspots where local conditions catalyzed diverse modes and pathways to change^4,5^?

Because domestication is a process that brings about changes at the level of populations, it may be impossible to pin it to a single location of origin^6,7^. Nor is this process unidirectional; it may include reversals, dead ends, interruptions, and multiple episodes of genetic introgression^7–9^. Like others^7–9^, we view the process of domestication as a continuum characterized by intensifying human-animal interactions. These range from controlling the movements of wild animals to selective culling, and ultimately, the selective breeding of animals in a captive environment.

Here, we explore the early stages of the domestication process when all animals retained the wild phenotype (a.k.a. morphologically wild). We use the term *managed* to distinguish morphologically wild animals under human control from *domesticated* individuals that have undergone phenotypic change as the result of long-term human intervention^9^. We focus especially on the lesser known, incipient part of the management process, before animals were selectively culled or bred. This scale of interaction is termed “game management” by Zeder^6^ or “control in the wild” by Vigne *et al*.^9^ and may include translocation or protection, restriction of movements, or food provisioning of animals by humans among other forms of human intervention. Important shifts in species representation may occur at the outset of the animal management process. They can be detected in the zooarchaeological record by increases in the relative abundance of animals with appropriate characteristics for domestication^10,11^. Other contextual information for the nascent management of one or a few species may come from changes in human hunting intensity (see below). Further intensification along the animal management continuum may manifest in the selective culling of animals and/or body-size diminution caused by the release of natural selective pressures or new living conditions.

Although the earliest dates for pig, cattle, sheep and goat management cluster in the northern Levant and southeastern Anatolia, evidence for incipient animal management has also begun to accumulate in other regions^12–15^. For example, high frequencies of juvenile and infant sheep in Levels 4–5 (ca. 10,400–10,100 cal. BP) at Aşıklı Höyük in Central Anatolia^15^ suggest that early human management of herd animals was coeval to or only slightly later than the generally accepted earliest evidence from Çayonü and Nevali Çori in southeastern Anatolia (10,500–10,000 cal. BP)^16,17^. Evidence from Cyprus shows that pigs were under some kind of human control even earlier, by 11,400 cal. BP, regardless of whether they were locally domesticated^9^. Uneven dating, both in regard to numbers of radiocarbon dates and contextual control, make chronological comparisons difficult across regions. However, refinements of some local chronologies testify to parallel rather than single-origin evolution of animal and plant management among regions of the Fertile Crescent (e.g.^18^, Figs S1 and S2 in^15^).

In the Jordan Valley and the Mediterranean Hills of the southern Levant, changes in the relative frequency, demographic profiles, stable isotope ratios, and body-sizes of goats have long hinted at an autochthonous process of goat management, albeit at a somewhat later date than elsewhere in Southwest Asia (10,000 cal. BP^19–21^). The appearance of non-native sheep in the Jordan Valley in the Mid PPNB (10,500–9,500 cal. BP) on the other hand, has led others to argue that the practice of managing caprines and the stock populations were introduced from adjoining regions^1,21^. Up to now, the question has been framed as an either-or dialectic. Either hypothesis might be correct, but we argue that a mix of these phenomena is far more likely.

A separate debate concerns the ecological background from which plant and animal management arose^22–25^. A long-standing hypothesis holds that animal management, whether locally invented or adopted from neighboring peoples, was a solution to resource intensification caused by population packing and/or limited mobility^14,26,27^. A purportedly alternative hypothesis states that domestication was the result of opportunistic enhancements by humans to locally food-rich habitats in the absence of population pressure or any other environmental stress^23,25^. In fact, these hypotheses involve very different scales of phenomena and are not mutually exclusive^24^. Moreover, sedentism itself will create local stresses irrespective of why people choose to settle down. What is more interesting to us is the interaction between local stresses and opportunities within a larger network of information exchange, of which the southern Levant was certainly part. Thus, we examine human hunting intensity in the periods leading up to animal management in the southern Levant. We then consider its relation to the circumstances in which management became important in the study area.

**Figure 1:**
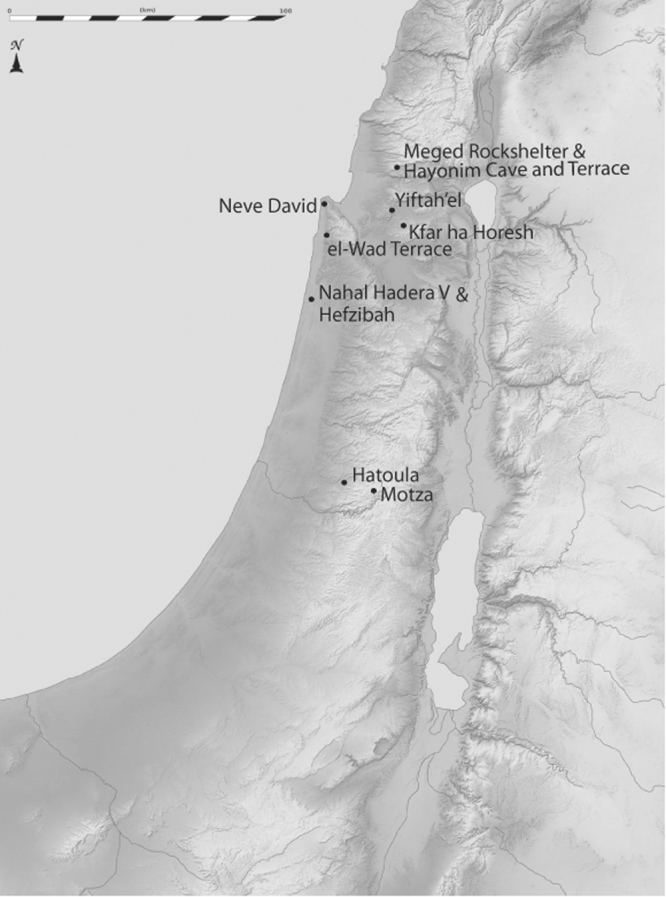
Map of sites in the study area (southern Levant). Map modified from image downloaded from Wikipedia commons: License: Eric Gaba (https://commons.wikimedia.org/wiki/User:Sting), Israel relief location map-blank (https://commons.wikimedia.org/wiki/File:Israel_relief_location_map-blank.jpg), color, added place names by Natalie Munro, CC BY-SA 3.0 (https://creativecommons.org/licenses/by-sa/3.0/legalcode).

Over the past 20 years, the authors have produced 16 large faunal databases from 11 sites which date to the Epipaleolithic through Pre-Pottery Neolithic B periods (Table 1 and Fig. 1). Our previous work has focused on the hunter-gatherers of the Late Pleistocene in the Mediterranean zone of the southern Levant, but the present compilation includes several new Pre-Pottery Neolithic (PPN) assemblages, enabling us to track change across the transition to agriculture for the first time. This high-resolution sequence allows us to examine competing hypotheses about the pathway(s) to ungulate management in this region (Table 2). Specifically, we use several lines of evidence to test the hypotheses that (a) ungulate management arose locally in the southern Levant, and (b) the process emerged from a background of intensive hunting. We test these hypotheses using data on taxonomic abundance, ungulate mortality and body-size. The data also provide more information on the pace of change.

**Table 1:** Sites, chronological period, site abbreviations, NISP and references for sites in the study sample.

Site	Period	Abbreviation	NISP	Reference
Yiftah’el	MPPNB	YIFT MPPNB	4229	^39^
Kfar haHoresh	MPPNB	KHH MPPNB	2737	^40^
Motza	MPPNB	MOTZ MPPNB	897	^41^
Motza	EPPNB	MOTZ EPPNB	6787	^41^
Hatoula	PPNA	HTLA PPNA	4706	Munro unpublished data
Hatoula	LNAT	HLTA LNAT	13189	Munro unpublished data
Hayonim Terrace	LNAT	HAYT LNAT	10132	^71^
Hayonim Cave	LNAT	HAYC LNAT	5692	^26,72^
el-Wad Terrace	LNAT	ELWT LNAT	1418	^47^
el-Wad Terrace	ENAT	ELWT ENAT	5819	^47^
Hayonim Cave	ENAT	HAYC ENAT	8909	^26,72^
Hefzibah	GKEB	HEFZ GKEB	8499	^72,73^
Neve David	GKEB	NVD KEB	2489	^72,73^
Nahal Hadera V	KEB	NHDR KEB	19295	^72,73^
Hayonim Cave	KEB	HAYC KEB	3575	^28^
Meged Rockshelter	KEB	MEGD KEB	1810	^28^
Meged Rockshelter	UP	MEDG UP	586	^28^
Hayonim Cave	UP	HAYC UP	9880	^28^
Hayonim Cave	MP	HAYC MP	15494	^28^

Cultural Periods are as follows MP = Middle Paleolithic, UP = Upper Paleolithic, KEB = Kebaran, GKEB = Geometric Kebaran, ENAT = Early Natufian, LNAT = Late Natufian, PPNA = Pre-Pottery Neolithic A, EPPNB = Early Pre-Pottery Neolithic B, MPPNB = Middle Pre-Pottery Neolithic B.

**Table 2:** Chronology of south Levantine cultural periods included in this study.

Chronology	Abbrev	Years cal bp
Middle Pre-Pottery Neolithic	MPPNB	10,100–9,500
Early Pre-Pottery Neolithic	EPPNB	10,500–10,100
Pre-Pottery Neolithic A	PPNA	11,600–10,500
Late Natufian	LNAT	13,600–11,600
Early Natufian	ENAT	15,000–13,600
Geometric Kebaran	GKEB	18,000–15,000
Kebaran	KEB	24,000–18,000
Upper Paleolithic	UP	~26,000–28,000
Middle Paleolithic 1/2	MP1/2	~70–100,000
Middle Paleolithic 3	MP3	~150,000
Middle Paleolithic 4	MP4	~170,000
Middle Paleolithic 5/6	MP5/6	~200,000

The chronology (calibrated years BP) follows Goring-Morris and Belfer-Cohen^29^. See Supplementary Table S1 for radiocarbon chronology for specific sites and cultural layers in the sample. The Paleolithic chronology is specific only to Hayonim Cave and follows Stiner^28^.

## Methods and Samples

In the southern Levant, the Epipaleolithic is represented by Kebaran, Geometric Kebaran and Natufian hunter-gatherer sites, while the Pre-Pottery Neolithic (PPN) includes the PPNA and the Early (E), and Middle (M) phases of the PPNB. Our sequence extends through the MPPNB (Tables 2 and S1; 9,500 cal. BP)^28,29^. For some analyses, we also include data from the Upper and Middle Paleolithic (Tables 2 and S1) to highlight the scope of change in the early Neolithic. The 11 archaeological sites are situated on the coastal plain near the edge of the Mediterranean Hills or in the Mediterranean Hills proper (Fig. 1). Restricting the study to these ecological zones helps to control for environmental variation that could bias prey selection and our perceptions of human living conditions during each period.

Evidence for the first stages of ungulate management are particularly elusive in the archaeological record. We begin by quantifying the relative abundance of prey taxa. This is helpful in research on the southern Levant, because we know that although humans hunted wild variants of goat (*Capra aegagrus*), wild boar (*Sus scrofa*) and wild cattle (*Bos primigenius*) at the end of the Pleistocene and the early Holocene, they were uncommon or rare^10,11,28,30^. The wild taxa that were most important in the meat diet prior to the Neolithic—mountain gazelle (*Gazella gazella*), Mesopotamian fallow deer (*Dama mesopotamica*) and red deer (*Cervus elaphus*)—were never domesticated.

We test the hypothesis that animal management replaced intensive hunting in the southern Levant by measuring human hunting intensity using a prey choice model^31^. Hunting intensity is defined as the amount of energy that humans invest in prey capture (including time) in relation to the energy gained from prey after handling costs are added^31^. As hunting intensifies, foraging efficiency declines and dietary breadth expands. Hunting is intensified most often in response to imbalances between consumer demand and prey returns. This imbalance can occur as the result of an increase in human population size at the regional scale or the local effects of sedentism. Either way, there will be a decrease in encounter rates with high-ranked prey due to local resource depression^32^ or climate-driven environmental change^33^. In the prey choice model^34^, potential prey species are ranked according to their post-encounter return rates. It is assumed that a forager will always choose the highest-ranked prey on encounter^31^. Lower-ranked prey items will be added to the diet if the overall return rate for the highest-ranked type declines to the point that it is equal to or less than the post-encounter return rate of the lower-ranked item^32^.

Trends in hunting intensity are evaluated using four indices of high- versus low-ranked game and mortality patterns in the assemblages from the Early Epipaleolithic through MPPNB periods^26,27,29^. Because the taxonomic groups investigated are distinguished by large differences in body-size, and thus different potential energetic returns, prey body-size serves as a proxy for differences in prey rank^32,35^. Given that food quality is similar among ungulate species but body weights differ, ungulate taxa are ranked in order of descending body-size—wild cattle (highest rank), red deer, fallow deer, pig, goat and gazelle (lowest rank)^27^. Common small game species such as the Mediterranean spur-thighed tortoise (*Testudo graeca*), hare (*Lepus capensis*), game birds (e.g., *Alectoris chukar*), and fish were also eaten, although they contributed less energetically. Small prey animals (Supplementary Table S2) are combined into a single small game category that is ranked lower than gazelle (but see^26,27^ in regard to tortoises).

The first index, based on the number of identified specimens (NISP), is the proportion of small ungulates (gazelles) of the total ungulates in each assemblage. The second index (NISP) is the proportion of small game animals relative to all taxa (including in ungulates and carnivores). The third index concerns the ages of the hunted gazelles as determined from bone fusion states, specifically the proportion of fawns (<6 months) and juveniles plus fawns (<18 months) in each assemblage. The proportion of unfused proximal 1^st^ and/or 2^nd^ phalanges (which fuse ca. 6 months of age) represents gazelle fawns, while the proportion of unfused distal metapodials (fuse ca. 18 months of age) represents the fawn and older juveniles combined^36,37^. The age-based measures are calculated from the minimum number of elements (MNE) to avoid double-counting of individuals. The proportion of young gazelles is taken to reflect hunting intensity, not only because juveniles are smaller-bodied and thus ranked lower than adults of the same species, but also because juveniles tend to become relatively more common in heavily hunted populations^38^. Shapiro-Wilk normality tests of our index data indicate that all samples met the conditions for normality prior to statistical comparisons using t-tests.

A final index based on NISP concerns the trade-off between wild prey and animals that were ultimately domesticated. This is a contrast between the relative representation of deer and gazelle versus cattle, pig and goat. For this final measure, we extend our chronological sequence back to the Middle Paleolithic period (ca. 200 kya) (Tables 2 and S1) to highlight changes in Neolithic ungulate composition, and contextualize them within long-term trends in the region.

If the hypothesis that intensive hunting was a formative background for ungulate management in the southern Levant is correct, then our relative abundance indices should signal intensive hunting in the cultural periods immediately prior to the trade-off between wild animals and cattle, pig and goat that could mark the beginning of a new human-animal relationship. The signatures of intensive hunting should reverse as the trade-off begins.

We test the hypothesis that animal management emerged locally in the southern Levant using a combination of ungulate relative abundance data and mortality and body-size data. Mortality profiles and body-size data from our Neolithic sites are synthesized from recent papers published by our team^39–41^. Mortality data based on tooth eruption and wear sequences and bone fusion for goats, cattle and pigs allow us to determine whether humans were manipulating reproduction and survivorship of the ungulates, as a delayed (managed meat and potentially milk) returns strategy. This strategy conserves the herd’s reproductive core (prime adult females) at the expense of juvenile males^42^. Because the domestication process often leads to body size reduction^43,44^, the Log Scale Index (following^45^) is used to detect changes in the average body-size of cattle, pig and goat by enabling the comparison of small samples of measurements.

If animal management emerged locally, we expect to see a gradual trade-off between wild game use (gazelle and deer) and at least some types of domesticate-able ungulates. At the very beginning of the trade-off, these locally available species need not show signs of selective culling of juvenile males^42,44,46^. In contrast, support for the null hypothesis that managed animals were adopted from the north would include the simultaneous appearance of sheep (a non-native ungulate) from the onset of the economic trade-off and evidence that cattle, pig and goat were already under selective culling.

### Data availability

All data is available in supplementary materials.

## Results

**Figure 2:**
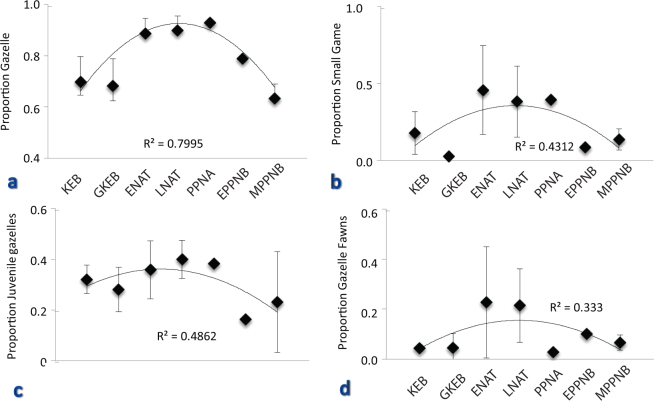
Relative abundance indices for the Epipaleolithic-PPN sequence. Trend lines are second order polynomial regression lines. (**a**) Proportion of gazelles out of all ungulates (includes gazelle, deer, cattle, goat, pig), raw data in Supplementary Table S3. (**b**) Proportion of small game (includes fish, tortoises, birds (except small perching birds) and hare) out of all taxa (ungulates, carnivores and small game), raw data in Supplementary Table S4; (**c**) proportion of juvenile gazelles (<18 mos) out of all gazelles, data in Supplementary Table S5; and (**d**) proportion of gazelle fawns (<6 mos) of all gazelles, data in Supplementary Table S5.

Figure 2a plots changes in the proportion of the lowest-ranked (smallest bodied) ungulate, the mountain gazelle, relative to all ungulates in each assemblage from the Epipaleolithic through the Neolithic periods. The proportion of gazelles in the later Natufian (NAT) and PPNA is significantly higher than in the Kebaran Geometric and Kebaran (t = 6.48, df = 5; p = 0.0007). By the Early Natufian, gazelles were nearly the only ungulate species hunted. Gazelle abundance levels off into the PPNA, declines in the EPPNB, and declines further in the MPPNB (difference between NAT&PPNA and E&MPPNB assemblages is highly significant; t = −5.308; df = 3; p = 0.006).

The small game index also increases significantly through the Epipaleolithic, peaking in the Natufian and PPNA periods (Fig. 2b; NAT&PPNA significantly greater than KEB&GKEB t = 2.98; df = 10; p = 0.007). The Natufian sites with the largest faunal middens are characterized by the highest proportions of low-ranked small game^26,27,47^, consistent with the hypothesis of consumer pressure on local resources. However, the trend in small game abundance reverses in the EPPNB (the beginning of a step-wise decline) and further in the MPPNB (E&MPPNB index significantly smaller than NAT&PPNA index; t = 1.86; df = 8; p = 0.005).

Figure 2c,d illustrates variation in the relative abundance of gazelle fawns (<6 months, Fig. 2c) and juveniles (<18 months, Fig. 2d) in the assemblages. Human exploitation of young gazelles increases most conspicuously from the Early Epipaleolithic to the Natufian (significant difference between KEB&GKEB and NAT&PPNA fawns; t = 2.45; df = 7; p = 0.02; and between KEB&GKEB and NAT&PPNA juveniles; t = 2.03; df = 10; p = 0.035). The abundance of fawns peaks in the Natufian, while exploitation of juvenile gazelles peaks in the PPNA. The proportion of both age groups declines back to early Epipaleolithic levels in the EPPNB and remains low through the MPPNB (significant difference between NAT&PPNA and E&MPPNB fawns; t = −1.92; df = 7; p = 0.05; and marginally insignificant difference between NAT&PPNA and E&MPPNB juveniles; t = −1.95; df = 4; p = 0.06).

Thus, the first shift in the relative abundance of high- and low-ranked game occurs in the Early Natufian, and the second shift coincides with the EPPNB. The same trajectory of hunting intensity—increase, plateau and decline—is apparent in all of the indices (small ungulate, small game and juvenile gazelle indices are significantly correlated; Table 3), except for the exploitation of fawns, which begin to drop in the PPNA (Table 3). These shifts in the patterns of animal exploitation do not correspond to notable episodes of climatic or environmental change^48^ over the time interval of our study (See Supplementary Note: Correlation with Climatic Change and Supplementary Fig. S1).

**Table 3:** Results from Spearman’s rank-order correlations of the four indices.

Index	Spearman’s rho; P value
Small Ungulate vs Small Game	**0**.**714**; **p** = **0**.**044**
Small Ungulate vs Juvenile Gazelle	**0**.**750**; **p** = **0**.**033**
Small Game vs Juvenile Gazelle	**0**.**750**; **p** = **0**.**033**
Small Ungulate vs Gazelle Fawn	0.071; p = 0.453
Small Game vs Gazelle Fawn	0.214; p = 0.331
Juvenile Gazelle VS Gazelle Fawn	0.038; p = 0.482

Three of the indices—small ungulate, small game and juvenile gazelle—significantly correlate with one another at the p < 0.05 level. The gazelle fawn index does not correlate with any of the other three indices.

**Figure 3:**
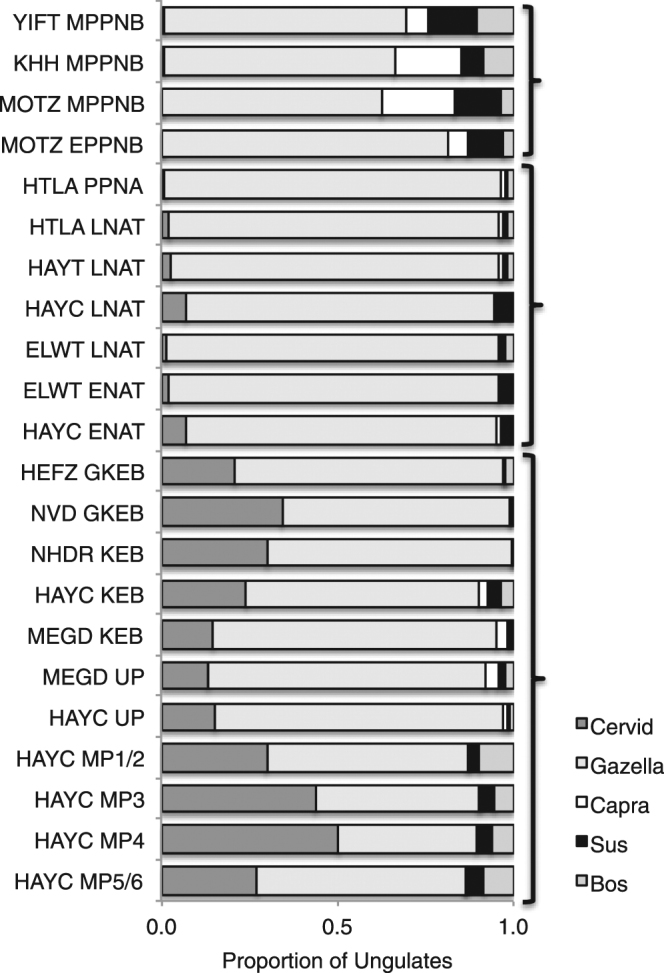
Relative abundance of primary ungulate taxa per site arranged in chronological order. Includes the Paleolithic sequence from Hayonim Cave to provide time depth. Ungulate taxa include cervids (roe deer, fallow deer and red deer), gazelle, goat, pig and cattle. See Tables 1 and 2 for site and cultural period abbreviations and Supplementary Table S3 for raw NISP values. Brackets indicate three groups of sites based on the similarity of their data: the lower bracket indicates pre-Natufian Epipaleolithic sites, the middle bracket encapsulates Natufian and PPNA sites and the upper bracket indicates PPNB sites.

Figure 3 includes data from earlier Upper Paleolithic (UP) and Middle Paleolithic (MP) assemblages from Hayonim Cave and Meged Rockshelter^27^ to illustrate near-categorical differences in ungulate representation between the Paleolithic, Epipaleolithic and the early Neolithic periods. Ungulate diversity was high in the early MP (Hayonim MP phases 5/6, 4, 3) but narrowed greatly through the Natufian. A Spearman’s rank-order correlation of Inverse of Simpson’s Diversity index values for each period shows a statistically significant decline in ungulate evenness from MP 5/6 through the Late Natufian (r = −0.833, p = 0.004). Large ungulates are the first to decline in the sequence, followed by medium ungulates^27^. Cattle, the largest ungulate species, peaks in the MP, but is nearly gone by the Geometric Kebaran. Medium-sized ungulates (mostly fallow deer, but also goat and pig) decline soon thereafter. What are left to hunters by the Natufian period are mainly mountain gazelles, the smallest of all of the ungulates.

Medium- and large-bodied ungulates do not resurge in faunal assemblages until the EPPNB. When they do, the dominant species are very different from those of the Paleolithic. Deer (cervids), second only to gazelle throughout the entire Paleolithic and Epipaleolithic sequence, virtually disappear from the Natufian and Neolithic diets. Instead, the larger-bodied ungulate taxa were goat, cattle and pig, all native to the Levant but much less abundant in earlier faunas. These taxa become important in the EPPNB, and their combined numbers increase further during the MPPNB. The results clearly show that wild ungulate taxa of the Natufian and PPNA diet were replaced in the EPPNB with the same large-bodied species that ultimately came under domestication (cattle, pig and goat). Importantly, the initial increase in cattle, pig and goat in the EPPNB corresponds with the initial decline in all indices of hunting intensity except the gazelle fawns, which drop slightly earlier. A second increase in cattle, pig and goat in the MPPNB corresponds to a second drop or continued low in all of the hunting intensity indices.

Mortality and body-size results for goat, cattle and pig from the Mediterranean Hill zone are only summarized here (see original results in^39–41^). Goat mortality data show that even by the MPPNB at Kfar haHoresh, high proportions of animals survived into adulthood (75% to 48 months of age^40^, and nearly as many were adults at Yiftah’el (62% survived beyond 36 months^39^). The results indicate that these herds were not selectively culled to optimize meat or milk production. Instead, the data best fit the mortality profile of a hunted population^49^. Interestingly, goat mortality data from MPPNB Abu Gosh (not included in this study), suggests a managed population as only 31% of the herd survived beyond 39 months of age^50^. Mortality patterns similar to those of Abu Gosh occur only later in our study sites—in the LPPNB at Kfar haHoresh when only 32% of goats survived beyond 48 months of age^40^, and during the Final PPNB/PPNC at Yiftah’el when only 33% of the goats lived past 42 months of age^13^. Nevertheless, the average body-size of the goat populations during the MPPNB at Kfar haHoresh and at Yiftah’el is significantly smaller than the goat population from EPPNB Motza (t = −2.647, df = 17, p = 0.007; t = 4.336, df = 26, p < 0.001 respectively based on LSI analysis^39,40^). These results indicate statistically significant diminution in goat body-size from the EPPNB to the MPPNB.

Cattle and pig mortality profiles from our Neolithic sites were constructed to the extent that sample sizes allow. Juveniles of both species show high rates of survivorship in the MPPNB. About 80% of cattle survived beyond 36 months at Yiftah’el^39^ and 66% beyond 48 months at Kfar haHoresh^40^. Seventy-five percent of the pigs lived past 24 months of age at Yiftah’el. The survivorship patterns for cattle and pig are consistent with hunted populations^48^. The cattle and pig populations also show a statistically significant decline in body size from the EPPNB to the MPPNB. The average body size of cattle from MPPNB Yiftah’el (n = 36) is significantly smaller (p < 0.001) than the EPPNB populations from Motza (see data and statistical results in^37^). The Kfar haHoresh MPPNB samples are too small to undertake this kind of analysis. Pig body size at Yiftah’el (n = 19) is significantly smaller (p < 0.001) than the population from EPPNB Motza (see data and statistical results in^39^).

Importantly, the statistically significant patterns of diminution in goats, cattle and pigs between EPPNB Motza and MPPNB Yiftah’el are not paralleled by diminution in mountain gazelles. Instead, the average measurements from the breadth of the glenoid fossa of the scapula (GB) of gazelles are statistically similar over time, while the breadth of the distal humerus (Bd) are significantly larger on average than at Yiftah’el than at Motza (for statistics and measurement data see Table S2 in^39^). The decline in average body-size occurs only for cattle, pig and goat, the three taxa that were ultimately domesticated. Wild mountain gazelles of the region underwent no such change.

## Discussion

### Did new human-animal relations emerge in the Mediterranean Hills of the southern Levant during the Pre-Pottery Neolithic

Our data indicate a reversal in the EPPNB of a long-term trend in intensive wild animal exploitation. The reversal takes the form of declines in small game, gazelles, and young gazelles in the faunal record. The intensity of bone processing also declines during this period at Motza^39^. All three of the indices drop further or remain low in the MPPNB. The stepwise declines in hunting intensity correspond directly to increases in the frequency of cattle, goats and pigs in the assemblages.

The increase in the relative abundance of medium- and large-bodied ungulates starting in the EPPNB is remarkable in energetic terms^15^. Low-ranked game drop out of the economic system after millennia of intensive hunting. This trade-off must reflect an increase in the accessibility of higher-ranked animals in the EPPNB. Yet red deer and fallow deer, ungulate species that were second only to gazelle in abundance during the Epipaleolithic periods, are barely represented by this period. The absence of high-ranked native deer in the later assemblages shows that the reduction in hunting pressure was not brought on by a resurgence of previously common ungulates (gazelle, fallow deer and red deer) on the landscape. As medium and large ungulate taxa resurge in EPPNB diets, the focus was on three previously (naturally) rare ungulate species—cattle, goat and pig. The characteristic that most clearly differentiates the two groups is how amenable the species were to domestication^11^.

The combined importance of cattle, goats and pig continued to grow into the MPPNB. Similar trends are noted, sometimes even more strongly, in the other MPPNB assemblages of the Mediterranean zone^50,51^. Four lines of evidence point to a new relationship—management—being forged between humans and herd animals in the Mediterranean Hills of the southern Levant. First, in the EPPNB this is expressed as: (1) an increased abundance of high-ranked goats, cattle and pigs and (2) a corresponding decline in human hunting intensity. That the addition of goat, pig and cattle to the early Neolithic diet was sufficient to release millennia of hunting intensity, provides a powerful signal for the onset of animal management. These two trends continue in the MPPNB when they are joined by (3) a statistically significant reduction in the average body-size of goat, pig and cattle but not gazelles, and (4) new culling strategies in the neighboring MPPNB site of Abu Gosh^50^, signaling the intensification of animal management practices.

### When did animal management first begin in the southern Levant

Our multiple faunal indices indicate that the manipulation of certain animal populations arose as early as the EPPNB (ca. 10,500 cal. BP) in the southern Levant. Evidence based on changes in relative taxonomic abundance and long-term trajectories of human hunting intensity reveal the very onset of the management process for the first time—500 years before new culling practices and body-size diminution first appear in the MPPNB record. In the northern Levant, demographic and morphological data already indicate full-fledged management by this time and thus the onset of management processes undoubtedly occurred even earlier there. The evidence for human control over pig populations from Cyprus has also been pushed back to 11,400 cal. BP^9^, through artificial introduction to the island, but these pig populations may never have developed into managed herds.

### Was the development of management provoked by pre-existing conditions of intensive hunting in the southern Levant

The faunal indices provide clear evidence for intensified hunting over the course of the Late Pleistocene and into the beginning of the Holocene^26,27^. Long-term declines in the relative abundance of large- and medium-bodied ungulates in descending order of body size provide early signals for resource depression on the local and regional scales in the southern Levant. As populations of larger taxa became less common on the landscape, hunters used lower-ranked taxa more—gazelles and small game. Additionally, other research has shown that among small game species, there was greater use of costly, fast-escape types with time^27^, another signal of declining foraging efficiency. Increased exploitation of young gazelles starting in the Early Natufian (15,000 cal. BP) also suggests resource depression, as human-induced mortality linked to increased sedentism pushed gazelle populations below carrying capacity and into a continuous growth mode^26^. Still other evidence for resource depression in the Natufian includes the decline in the body-size of the Mediterranean spur-thighed tortoise, a species very sensitive to over-harvesting^27^, and intensified ungulate bone processing for marrow and grease^52^.

By the Natufian period (15,000 cal. BP), foraging efficiency had dropped so much that resources with higher handling costs (small game) became major components of the meat diet^26^. Broadening the diet in this way dramatically increased the potential yield per unit land area and therefore local carrying capacity. Intensive hunting practices continued into the PPNA (11,600 cal. BP). Plant cultivation in this period meanwhile increased the reliability and productivity of low-ranked grasses such as barley, wheat^53^ and other seeds^54,55^, which fortified human commitments to sedentism^56,57^. Our data show that the pressures on natural meat supplies were not relieved by plant cultivation. Humans continued to inhabit small permanent settlements and obtained animal resources by intensively hunting wild prey. They were clearly squeezing these wild prey populations, and sometimes depressing their abundance on the landscape.

Resource depression was caused by humans, particularly as they become more sedentary. Sustained hunting in the vicinity of habitation sites would have lowered encounter rates and increased search times for preferred large prey^58,59^. A continued focus on quick small game entailed higher capture costs^26^, but provided a more sustainable source of animal products because many of these species are also very productive (hares, ground birds). At some point, a threshold was crossed, when it became more cost effective to manipulate certain animal populations than to continue to depend on wild resources exclusively, not least because people were increasingly tethered to an agricultural lifestyle^60^.

Our data reveal a clear historical connection between hunting intensification and emergent human management of ungulates in the Mediterranean Hills of the southern Levant—a classic example of a strategic economic trade-off. Importantly, the stepwise drops in hunting intensity correspond directly to increases in the frequency of cattle, goats and pigs among the ungulate remains in the faunal assemblages. Thus, the trade-off appears to have been a solution to an impoverished hunting regime characterized by high search costs. These costs were likely inflated by the well-documented trend toward sedentism^26,47,57^. Related factors may also have been in play on account of the growing dependence on cereals^53^, such as territorial circumscription^61,62^ and/or local population growth^27^.

### Did early management of ungulates in the southern Levant have a local origin, an extra-local origin, or did it arise from an interactive combination of these sources

Our data show that the surprising rise of goats, pigs and cattle in early Neolithic faunas was not a simple case of importation. Three lines of zooarchaeological evidence suggest a predominantly (but not exclusively) local population contribution to the management process.

An important contradiction to simple importation of livestock and husbandry methods from the north is the absence of sheep at the time when goats, cattle and pigs were increasing rapidly in the study area. The latter three species naturally occur in the southern Levant, but wild sheep do not^63,64^. Both sheep and goats came under management quite early in areas to the north^10,11,65^. If early managed animals were simply imported from the north, then sheep should have been among them. A few sheep have been identified in the MPPNB layers at Jericho and Ain Ghazal^11,19,21^, but they are extraordinarily rare, and none have been identified in the Mediterranean Hills sites.

A second contradiction to simple importation of livestock and husbandry methods from the north is the absence of reproductive control or selective culling of younger male goats at EPPNB Motza in the interval when their frequencies first rise. The EPPNB animals appear to have been morphologically wild, local forms, as there is no evidence of morphological change at this time. The evidence instead suggests that the change in human-animal relationships in the EPPNB involved local ungulate populations to a significant degree.

Goats provide the most compelling evidence for an initially local management process. Goat relative abundance increases first in the EPPNB, and again in the MPPNB. New culling strategies and body-size diminution are also evidenced at some MPPNB sites (see above and^13,39,40,50^). As might be expected for a period of economic flux and experimentation, the goat demographic patterns are highly variable among MPPNB sites in the Mediterranean Hills. These observations do not fit the idea of wholesale importation of managed or domesticated animals into the region^10,13,14,39,50^.

Although, our data cannot speak conclusively to livestock importation after the EPPNB—it is possible that managed animals were imported to the southern Levant in the MPPNB and later. Still, our evidence suggests that the balance of local and imported animals was biased toward the former. It is very likely that “diffusion” of managed animals among regions within the Fertile Crescent required significant introgression with local reservoirs of wild variants in order to maintain genetic viability^8^. However, wild variants were already in the early stages of management in the southern Levant. The critical importance of local gene pools for building viable herds may actually mask the early stages of local management in the southern Levant, but this question must be explored through further work on ancient DNA.

The surprising increase in pig and cattle frequencies in the EPPNB suggests that people may have experimented in similar ways with the management of pigs and cattle. Our samples for these species are more limited, but we found no evidence for selective culling of cattle or pig in the EPPNB or MPPNB occupations at Yiftah’el, Motza and Kfar haHoresh. Data from the Jordan Valley suggest that selective culling did not begin until the PPNC or Pottery Neolithic (PN) for cattle and the LPPNB through PN for pig^14,66^. Nevertheless, the body-size data from Motza, Kfar haHoresh and Yiftah’el demonstrate statistically significant decreases in body-size as early as the MPPNB^40,66^. Overall, the story for pig and cattle management in the Mediterranean Hills seems to be more protracted than for goats, although more data are necessary to test this interpretation.

## Conclusions

The faunal data used in this study expose a deeper history for herd animal management evolution in the southern Levant. The trajectory was quite different to that of the northern areas of the Fertile Crescent, but the evidence for an *in situ* emergence of goat management is strong enough to force a reconsideration of common narratives for the southern Levant. Horwitz^10,13^ in particular has argued for local goat domestication. Earlier studies have been provocative on this question. Our new results are based on larger, more comprehensive datasets and also point to an earlier incipient process of early management of local ungulate (especially goat) populations. Subsequent importation of managed or domesticated populations to replace or bolster local animals in later phases of the Neolithic cannot be excluded, and seems likely. The evidence for pig and cattle may also suggest early manipulation by humans^14,66,67^. Sheep, which were managed by humans quite early in the northern areas of Fertile Crescent, were not part of the EPPNB strategies in the southern Levant. Their absence in the PPNA and EPPNB of our study area may be explained by the lack of a supporting wild reservoir for developing viable herds.

The southern Levant represents one of several regional pathways to ungulate management^7,9^. PPN groups are known to have tapped into wide social networks, through which knowledge and materials circulated among regions. These networks were particularly well developed by the PPNB^68,69^, as evidenced by the exchange of geographically restricted materials such as obsidian, marine shells and greenstone^70^. Unique seed-stock and management know-how undoubtedly were also transferred over large distances. Under these conditions, many changes in human practices could evolve in parallel, making economic reorganization and incipient management an overwhelmingly local process, even if it was ultimately seeded with small amounts of imported stock. PPN communities were rather insular in some regards, judging from the diversity of architectural traditions, but drew from extra-local knowledge whenever local conditions required or encouraged it. These opportunities for innovation were counter-balanced by the unique ecologies and cultural histories of the different parts of the Fertile Crescent, resulting in distinct local evolutionary pathways toward the early management of plants and animals.

## Electronic supplementary material

Supplementary Information 
