# Supplementary material for: The Emergence of Animal Management in the Southern Levant
Source: Sci Rep. 2018 Jun 18;8:9279. doi: 10.1038/s41598-018-27647-z (PMC6006362; doi:10.1038/s41598-018-27647-z)
Supplement: Supplementary file 1 — Supplementary Information [file 41598_2018_27647_MOESM1_ESM.doc]

**The Emergence of Animal Management in the Southern Levant**

Natalie D. Munro^a*^, Guy Bar-Oz^b^, Jacqueline S. Meier^c^, Lidar Sapir-Hen^d^, Mary C. Stiner^e^ and Reuven Yeshurun^b^

^a^Department of Anthropology, Unit 1176, 354 Mansfield Road, University of Connecticut, Storrs, CT 06250, USA

^b^Zinman Institute of Archaeology, University of Haifa, Mount Carmel, 31905 Haifa, Israel

^c^Department of Anthropology, Trent University, DNA Block C, 2140 East Bank Drive,
Peterborough, ON, K9J 7B8, Canada

^d^Department of Archaeology and Ancient Near Eastern Cultures, Tel Aviv University, Tel Aviv 6997801, Israel

^e^School of Anthropology, P.O. Box 210030, University of Arizona, Tucson, AZ 85721-0030, USA

^*^to whom correspondence should be addressed: Department of Anthropology, Unit 1176, 354 Mansfield Road, University of Connecticut, Storrs, CT 06250. Email: natalie.munro@uconn.edu

**Supplementary Notes**

**Supplementary Note: Correlation with Climatic Change**

Our indices of hunting intensity crosscut significant climatic fluctuations during our study period (Supplementary Fig. S1 online). Supplemental Figure S1 presents the Late Pleistocene-Early Holocene climatic record derived from carbon and oxygen isotope proxies measured in speleothems from Soreq (data from Bar-Matthews et al. 1999; 48). <ftp://ftp.ncdc.noaa.gov/pub/data/paleo/speleothem/israel/soreq_peqiin_2003.txt)>. Soreq Cave is located less than 15 km from two of our study sites (Motza and Hatoula) in the Mediterranean Zone of Central Israel. The oxygen isotope values provide an established regional proxy for temperature—high values mark arid years, while low values indicate wetter years. The carbon values provide a proxy for the precipitation available to the plants growing above Soreq Cave at the time of speleothem formation—negative carbon isotope values indicate higher rainfall than more positive values (Bar-Matthews et al. 2003).

  The patterns in our data do not correspond to the climatic data provided by the Soreq speleothems. Peak hunting intensity is reached in the Early Natufian and remains steady across the Late Natufian and PPNA. However, this timeframe is marked by significant climatic instability—conditions shifted rapidly from peak warm and wet conditions in the Early Natufian, to cooler temperatures typical of the Younger Dryas in the corresponding Late Natufian phase (Hartman et al., 2016), before reverting back to warm and wet conditions in the PPNA (supplementary materials Fig S2 online; Bar-Matthews et al. 1999;48). Likewise, periods of low hunting intensity correspond to a wide range of climatic conditions, including the cool Late Glacial Maximum that falls within the Kebaran period, a post-glacial warming trend during the Geometric Kebaran, and the warm and wet conditions of the Early Holocene that correspond to the E-MPPNB (supplementary materials Fig S2 online; Bar-Matthews et al. 1999; 48).

  Nor can other environmental changes such as potential land clearance in the PPN be responsible for the patterns observed here. Land clearance would favor open, grassland ungulates such as gazelle, but the E-MPPNB is marked by gazelle decline rather than expansion. The trajectories of our independent indices correlate significantly with one another (Table 3) and not to larger patterns of climate and/or environmental change.

Reference

Bar-Matthews, M., Ayalon, A., Kaufman, A. and Wasserburg, G. The Eastern Mediterranean paleoclimate as a reflection of regional events: Soreq cave, Israel. *Earth Planet Sci Let*, **166**(1-2), 85-95 (1999).

Hartman, G., et al. Hunted gazelles evidence cooling, but not drying, during the Younger Dryas in the southern Levant. *P Natl Acad Sci USA* **113**(15), 3997-4002 (2016).

**Supplementary Figure**

Supplementary Figure S1: Oxygen and carbon stable isotope data derived from speleothems from Soreq Cave located in the Mediterranean zone of Central Israel provide climatic proxies for precipitation and aridity. The graph was created from published isotope data from Bar-Matthews et al. (43; <ftp://ftp.ncdc.noaa.gov/pub/data/paleo/speleothem/israel/soreq_peqiin_2003.txt>). The trendlines were smoothed using a moving average, each period represents an average of five measurements. Dashed vertical lines and the numbers bounded by these lines represent the cultural periods investigated in this study. Major climatic downturns including the Last Glacial Maximum (LGM) and the Younger Dryas (YD) are indicated by light shading. High oxygen values represent cooler temperatures, while lower values indicate warmer temperatures. High carbon values mark periods of aridity, while low carbon values indicate wetter periods. Figure drafted by Gideon Hartman.

**Supplementary Tables**

Supplementary Table S1: Radiocarbon chronology for study assemblages.

| Site | Cultural  Period | Date in  Cal. BP (95%) | n C14 Dates | Reference |
| --- | --- | --- | --- | --- |
| Yiftah'el | MPPNB | *No C14 Dates | 0 | N/A |
| Kfar haHoresh | MPPNB | Dates unpublished | 0 | N/A |
| Motza | MPPNB | 9,170-8,590 | 4 | 1 |
| Motza | EPPNB | 10,550-9,900 | 12 | 2 |
| Hatoula | PPNA | 12,240-9,690 | 3 | 3 |
| Hatoula | LNAT | 13,150-12,646 | 1 | 3 |
| Hayonim Terrace | LNAT | 13,940-12,940 | 5 | 4 |
| Hayonim Cave | LNAT | No C14 Dates | 0 | N/A |
| el-Wad Terrace | LNAT | 13,720–11,610 | 5 | 5 |
| el-Wad Terrace | ENAT | 14,850-13,180 | 13 | 5, 6 |
| Hayonim Cave | ENAT | 15,030-13,570 | 2 | 7 |
| Hefzibah | GKEB | No C14 Dates | 0 | N/A |
| Neve David | GKEB | No C14 Dates | 0 | N/A |
| Nahal Hadera | KEB | No C14 Dates | 0 | N/A |
| Hayonim Cave | KEB | No C14 Dates | 0 | N/A |
| Meged Rockshelter | KEB | 22,310-21,550 | 2 | 8 |

Key for abbreviations of cultural periods in caption of Table 1.

The ^14^C date ranges from each site have been calibrated with CALPAL-2007 and the CALPAL-2007 HULU curve (CalPal-2007, Cologne Radiocarbon Calibration & Palaeoclimate Research Package, Radiocarbon Lab Köln. <http://www.calpal.de/>). The ranges were obtained from calibrating the set of dates from each site.

**References Cited in Supplementary Table S1**

1. Boaretto, E., & Khalaily, H. Dating the Pre Pottery Neolithic B of Motza in *Prehistoric Motza*, (eds. Khalaily, H. & Rose, A.) (lsrael Antiquities Authority Reports, forthcoming).

2. Khalaily, H. et al. Excavations at Motza in the Judean Hills and the Early Pre-Pottery Neolithic B in the Southern Levant. *Paléorient* **33**(2), 5-38 (2007).

3. Valladas, H., & Arnold, M. Datation 14C par accélérateur de site de Hatoula in *Le site de Hatoula en Judée occidentiale, Israël* (eds. Lechevallier, M. & Ronen, A.) 35-36 (Mémoires et Travaux du Centre de Recherche Français de Jérusalem 8, 1994).

4. Housley, R. Eastern Mediterranean chronologies: the Oxford AMS contribution in *Late Quaternary Chronology and Palaeoclimates of the Eastern Mediterranean* (eds. Bar-Yosef, O., & Kra, R.) 55-73 (Radiocarbon, 1994).

5. Caracuta, V. et al. Charred wood remains in the Natufian sequence of el-Wad terrace (Israel): New insights into the climatic, environmental and cultural changes at the end of the Pleistocene. *Quaternary Sci Rev* **131**, 20-32 (2016).

6. Weinstein-Evron, M. et al. New ^14^C dates for the Early Natufian of el-Wad Terrace, Mount Carmel, Israel. *Radiocarbon* **54**, 813-822 (2012).

7. Bar-Yosef, O., The archaeology of the Natufian layer at Hayonim Cave in *The Natufian culture in the Levant* (eds. Valla, F & Bar-Yosef, O.) 81-92 (International Monographs in Prehistory, 1991).

8. Kuhn, S. et al. The Last Glacial Maximum at Meged Rockshelter, Upper Galilee, Israel. *Journal of the Israel Prehistoric Society* **34**, 5-47 (1994).

Supplementary Table S2: Raw NISP Data for all study assemblages included here. General taxonomic categories other than ungulate body-size categories excluded. Only small game certain to have been exploited by humans included.

|  | **HAYC** | **HAYC** | **HAYC** | **HAYC** | **HAYC** | **MEGD** | **MEGD** | **HAYC** | **NHDR** | **NVD** | **HEFZ** |
| --- | --- | --- | --- | --- | --- | --- | --- | --- | --- | --- | --- |
| **TAXON** | **MP 1/2** | **MP 3** | **MP 4** | **MP 5/6** | **UP** | **UP** | **KEB** | **KEB** | **KEB** | **GKEB** | **GKEB** |
| **Ungulates** |  |  |  |  |  |  |  |  |  |  |  |
| *Capreolus capreolus* | 1 | 7 | 2 | 1 | 77 | 0 | 1 | 16 | 17 | 26 | 0 |
| *Dama mesopotamica* | 34 | 156 | 738 | 234 | 798 | 16 | 58 | 198 | 5414 | 780 | 1685 |
| *Cervus elaphus* | 11 | 24 | 92 | 26 | 236 | 6 | 10 | 72 | 0 | 8 | 0 |
| Cervidae | 7 | 44 | 183 | 83 | 51 | 5 | 14 | 93 | 0 | 0 | 0 |
| *Gazella gazella* | 101 | 244 | 792 | 746 | 6253 | 160 | 453 | 1039 | 12528 | 1540 | 6169 |
| *Capra aegagrus* | 1 | 4 | 1 | 1 | 103 | 7 | 19 | 36 | 0 | 0 | 0 |
| *Bos primigenius* | 17 | 29 | 127 | 105 | 77 | 5 | 1 | 53 | 43 | 13 | 212 |
| *Sus scrofa* | 5 | 20 | 84 | 67 | 52 | 4 | 7 | 63 | 10 | 9 | 11 |
| *Equus* sp. | 0 | 5 | 11 | 6 | 3 | 1 | 1 | 3 | 100 | 0 | 1 |
| *Alcephalus busephalus* | 0 | 0 | 0 | 0 | 12 | 0 | 0 | 0 | 154 | 6 | 7 |
| *Diocerorhinus hemitoechus* | 0 | 0 | 2 | 0 | 1 | 0 | 0 | 0 | 0 | 0 | 0 |
| Small Ungulate | 105 | 268 | 1006 | 589 | ND | 131 | 427 | 722 | ND | ND | ND |
| Medium Ungulate | 54 | 335 | 1694 | 550 | ND | 89 | 210 | 537 | ND | ND | ND |
| Large Ungulate | 28 | 69 | 192 | 166 | ND | 6 | 14 | 110 | ND | ND | ND |
| **Carnivores** |  |  |  |  |  | **0** |  |  |  |  |  |
| *Felis sp.* | 2 | 0 | 2 | 5 | 67 | 2 | 1 | 6 | 15 | 0 | 7 |
| *Canis* sp*.* | 0 | 0 | 4 | 1 | 1 | 0 | 0 | 1 | 5 | 10 | 0 |
| *Vulpes vulpes* | 3 | 7 | 8 | 0 | 147 | 2 | 6 | 9 | 190 | 41 | 105 |
| *Meles meles* | 0 | 0 | 0 | 0 | 0 | 0 | 0 | 0 | 0 | 0 | 0 |
| *Martes foina* | 0 | 1 | 2 | 0 | 5 | 0 | 4 | 0 | 0 | 1 | 0 |
| *Vormela peregusna* | 0 | 0 | 0 | 0 | 0 | 0 | 0 | 0 | 0 | 0 | 0 |
| *Herpestes ichneumon* | 0 | 0 | 0 | 0 | 0 | 0 | 0 | 0 | 0 | 3 | 0 |
| *Panthera sp.* | 0 | 0 | 8 | 1 | 0 | 0 | 0 | 3 | 0 | 0 | 0 |
| *Lycaon sp.* | 0 | 0 | 0 | 4 | 0 | 0 | 0 | 0 | 0 | 0 | 0 |
| *Hyaenidae* | 0 | 0 | 0 | 0 | 1 | 0 | 0 | 0 | 0 | 0 | 0 |
| *Ursus arctos* | 1 | 3 | 3 | 14 | 0 | 0 | 0 | 2 | 0 | 0 | 0 |
| **Small Game** |  |  |  |  |  |  |  |  |  |  |  |
| Pices (Indeterminant) | 0 | 0 | 0 | 0 | 0 | 0 | 0 | 0 | 0 | 0 | 0 |
| *Testudo graeca* | 62 | 426 | 3155 | 2601 | 1118 | 124 | 462 | 453 | 320 | 12 | 60 |
| Medium Bird | 4 | 15 | 21 | 17 | 0 | 11 | 62 | 29 | 16 | 0 | 3 |
| Large Bird | 2 | 9 | 7 | 8 | 572 | 4 | 0 | 16 | 0 | 0 | 0 |
| Huge Bird | 2 | 9 | 7 | 0 | 0 | 2 | 0 | 11 | 0 | 0 | 0 |
| Indet. Birds | 1 | 0 | 5 | 0 | 306 | 11 | 35 | 91 | 9 | 0 | 0 |
| *Lepus capensis* | 2 | 3 | 0 | 0 | 0 | 0 | 25 | 12 | 474 | 39 | 239 |
| *Hystrix indica* | 0 | 0 | 0 | 0 | 0 | 0 | 0 | 0 | 0 | 1 | 0 |
| **TOTAL** | **443** | **1678** | **8148** | **5225** | **9880** | **586** | **1810** | **3575** | **19295** | **2489** | **8499** |

Supplementary Table S2 Continued

|  | **HAYC** | **ELWT** | **ELWT** | **HAYC** | **HAYT** | **HTLA** | **HTLA** | **MOTZ** | **MOTZ** | **KHH** | **YIFT** |
| --- | --- | --- | --- | --- | --- | --- | --- | --- | --- | --- | --- |
| **TAXON** | **EN** | **EN** | **LN** | **LN** | **LN** | **LN** | **PPNA** | **EPPNB** | **MPPNB** | **MPPNB** | **MPPNB** |
| **Ungulates** |  |  |  |  |  |  |  |  |  |  |  |
| *Capreolus capreolus* | 5 | 2 | 1 | 1 | 26 | 57 | 11 | 2 | 1 | 1 | 0 |
| *Dama mesopotamica* | 47 | 24 | 3 | 30 | 90 | 49 | 7 | 3 | 0 | 2 | 0 |
| *Cervus elaphus* | 23 | 0 | 0 | 13 | 19 | 7 | 3 | 0 | 0 | 0 | 1 |
| Cervidae | 41 | 0 | 0 | 27 | 13 | 0 | 0 | 0 | 0 | 2 | 18 |
| *Gazella gazella* | 1496 | 1068 | 300 | 883 | 5008 | 4512 | 1785 | 2881 | 289 | 568 | 1974 |
| *Capra aegagrus* | 17 | 0 | 0 | 8 | 35 | 45 | 23 | 201 | 96 | 166 | 182 |
| *Bos primigenius* | 11 | 5 | 7 | 4 | 93 | 76 | 31 | 100 | 18 | 77 | 304 |
| *Sus scrofa* | 50 | 42 | 6 | 43 | 85 | 67 | 13 | 358 | 60 | 50 | 383 |
| *Equus* sp. | 0 | 0 | 0 | 1 | 0 | 0 | 0 | 0 | 0 | 0 | 0 |
| *Alcephalus busephalus* | 0 | 0 | 0 | 0 | 0 | 9 | 0 | 0 | 0 | 0 | 0 |
| *Diocerorhinus hemitoechus* | 0 | 0 | 0 | 0 | 0 | 0 | 0 | 0 | 0 | 0 | 0 |
| Small Ungulate | 1018 | 2873 | 697 | 574 | 123 | 5894 | 341 | 1155 | 88 | 831 | 59 |
| Medium Ungulate | 87 | 88 | 7 | 52 | 108 | 106 | 61 | 298 | 50 | 194 | 642 |
| Large Ungulate | 22 | 6 | 1 | 10 | 17 | 62 | 23 | 50 | 9 | 111 | 54 |
| **Carnivores** |  |  |  |  |  |  |  |  |  |  |  |
| *Felis sp.* | 54 | 25 | 3 | 51 | 52 | 13 | 45 | 154 | 18 | 65 | 34 |
| *Canis* sp*.* | 3 | 20 | 0 | 3 | 31 | 9 | 3 | 5 | 3 | 1 | 7 |
| *Vulpes vulpes* | 116 | 186 | 43 | 153 | 373 | 141 | 439 | 973 | 106 | 162 | 316 |
| *Meles meles* | 13 | 11 | 2 | 20 | 21 | 27 | 8 | 4 | 2 | 0 | 2 |
| *Martes foina* | 17 | 19 | 6 | 15 | 8 | 3 | 50 | 17 | 5 | 1 | 10 |
| *Vormela peregusna* | 12 | 1 | 0 | 5 | 7 | 11 | 9 | 0 | 0 | 5 | 0 |
| *Herpestes ichneumon* | 0 | 2 | 0 | 0 | 0 | 1 | 0 | 0 | 0 | 0 | 4 |
| *Panthera sp.* | 0 | 0 | 0 | 0 | 0 | 0 | 0 | 1 | 0 | 0 | 0 |
| *Lycaon sp.* | 0 | 0 | 0 | 0 | 0 | 0 | 0 | 0 | 0 | 0 | 0 |
| *Hyaenidae* | 0 | 0 | 0 | 0 | 0 | 0 | 0 | 0 | 0 | 0 | 0 |
| *Ursus arctos* | 0 | 0 | 0 | 0 | 0 | 0 | 0 | 0 | 0 | 0 | 0 |
| **Small Game** |  |  |  |  |  |  |  |  |  |  |  |
| Pices (Indeterminant) | 23 | 0 | 0 | 8 | 23 | 2 | 174 | 0 | 0 | 3 | 0 |
| *Testudo graeca* | 1777 | 1134 | 303 | 2542 | 2987 | 1447 | 277 | 313 | 136 | 245 | 137 |
| Medium Bird | 2273 | 60 | 8 | 715 | 166 | 41 | 461 | 0 | 0 | 14 | 15 |
| Large Bird | 215 | 6 | 0 | 99 | 43 | 18 | 233 | 0 | 0 | 6 | 30 |
| Huge Bird | 30 | 3 | 0 | 18 | 8 | 0 | 0 | 0 | 0 | 3 | 2 |
| Indet. Birds | 0 | 13 | 1 | 0 | 56 | 389 | 0 | 92 | 9 | 5 | 2 |
| *Lepus capensis* | 1559 | 231 | 30 | 417 | 740 | 203 | 709 | 180 | 7 | 225 | 53 |
| *Hystrix indica* | 0 | 0 | 0 | 0 | 0 | 0 | 0 | 0 | 0 | 0 | 0 |
| **TOTAL** | **8909** | **5819** | **1418** | **5692** | **10132** | **13189** | **4706** | **6787** | **897** | **2737** | **4229** |

Supplementary Table S3: NISP values for major ungulate taxa represented in the study sites (Fig. 3) and %*Gazella* of these combined ungulate taxa (Fig, 2a).

| SITE | PERIOD | *Gazella* | Cervidae | *Capra* | *Bos* | *Sus* | Total | %*Gazella* |
| --- | --- | --- | --- | --- | --- | --- | --- | --- |
| Yiftah'el | MPPNB | 1974 | 19 | 182 | 304 | 383 | 2862 | 69.0 |
| Kfar ha Horesh | MPPNB | 568 | 5 | 166 | 77 | 50 | 866 | 65.6 |
| Motza | MPPNB | 289 | 1 | 96 | 18 | 60 | 464 | 62.3 |
| Motza | EPPNB | 2881 | 5 | 201 | 100 | 358 | 3545 | 81.3 |
| Hatoula | PPNA | 1785 | 21 | 23 | 31 | 13 | 1873 | 95.3 |
| Hatoula | LNAT | 4512 | 113 | 45 | 76 | 67 | 4813 | 93.7 |
| Hayonim Terrace | LNAT | 5008 | 148 | 35 | 93 | 85 | 5369 | 93.3 |
| Hayonim Cave | LNAT | 883 | 71 | 8 | 4 | 43 | 1009 | 87.5 |
| el-Wad Terrace | LNAT | 300 | 4 | 0 | 7 | 6 | 317 | 94.6 |
| el-Wad Terrace | ENAT | 1068 | 26 | 0 | 5 | 42 | 1141 | 93.6 |
| Hayonim Cave | ENAT | 1496 | 116 | 17 | 11 | 50 | 1690 | 88.5 |
| Hefzibah | GKEB | 6169 | 1685 | 0 | 212 | 11 | 8077 | 76.4 |
| Neve David | GKEB | 1540 | 814 | 0 | 13 | 9 | 2376 | 64.8 |
| Nahal Hadera V | KEB | 12528 | 5431 | 0 | 43 | 10 | 18012 | 69.6 |
| Hayonim Cave | KEB | 1039 | 379 | 36 | 53 | 63 | 1570 | 66.2 |
| Meged Rockshelter | KEB | 453 | 83 | 19 | 1 | 7 | 563 | 80.5 |
| Meged Rockshelter | UP | 160 | 27 | 7 | 5 | 4 | 203 | 78.8 |
| Hayonim Cave | UP | 6253 | 1162 | 103 | 77 | 52 | 7647 | 81.8 |
| Hayonim Cave | MP1/2 | 101 | 53 | 1 | 17 | 5 | 177 | 57.1 |
| Hayonim Cave | MP3 | 244 | 231 | 4 | 29 | 20 | 528 | 46.2 |
| Hayonim Cave | MP4 | 792 | 1015 | 1 | 127 | 84 | 2019 | 39.2 |
| Hayonim Cave | MP5/6 | 746 | 344 | 1 | 105 | 67 | 1263 | 59.1 |

Supplementary Table S4: NISP of small game, total assemblage and %small game in each assemblage (Fig. 2b).

| Site | Period | Small Game | Total | %Small Game |
| --- | --- | --- | --- | --- |
| Yiftah'el | MPPNB | 239 | 4229 | 5.7 |
| Kfar haHoresh | MPPNB | 501 | 2737 | 18.3 |
| Motza | MPPNB | 152 | 897 | 16.9 |
| Motza | EPPNB | 585 | 6787 | 8.6 |
| Hatoula | PPNA | 1854 | 4706 | 39.4 |
| Hatoula | LNAT | 2100 | 13189 | 15.9 |
| Hayonim Terrace | LNAT | 4023 | 10132 | 39.7 |
| Hayonim Cave | LNAT | 3799 | 5692 | 66.7 |
| el-Wad Terrace | LNAT | 342 | 1418 | 24.1 |
| el-Wad Terrace | ENAT | 1447 | 5819 | 24.9 |
| Hayonim Cave | ENAT | 5877 | 8909 | 66 |
| Hefzibah | GKEB | 302 | 8499 | 3.6 |
| Neve David | GKEB | 52 | 2489 | 2.1 |
| Nahal Hadera V | KEB | 819 | 19295 | 4.2 |
| Hayonim Cave | KEB | 612 | 3575 | 17.1 |
| Meged Rockshelter | KEB | 584 | 1810 | 32.3 |

Supplementary Table S5: Percent unfused gazelle first and second phalanges and metapodials (Fig. 2c and 2d).

| Site | Period | UF P1&P2 | P1&P2 | %UF P1&P2 | N UF MP | MP | %UF MP |
| --- | --- | --- | --- | --- | --- | --- | --- |
| Yiftah'el | MPPNB | 8* | 148* | 5.4* | 8 | 156 | 5.1 |
| Kfar haHoresh | MPPNB | 4* | 40* | 10.0* | 8 | 18 | 44.4 |
| Motza | MPPNB | 3 | 70 | 4.3 | 1 | 5 | 20.0 |
| Motza | EPPNB | 45 | 443 | 10.2 | 21 | 129 | 16.3 |
| Hatoula | PPNA | 2 | 71 | 2.8 | 15.5 | 40.5 | 38.3 |
| Hatoula | LNAT | 54* | 348* | 15.5* | 115 | 238 | 48.3 |
| Hayonim Terrace | LNAT | 31 | 186 | 16.7 | 51 | 127 | 40.2 |
| Hayonim Cave | LNAT | 31 | 72 | 43.1 | 19 | 46 | 41.3 |
| el-Wad Terrace | LNAT | 8* | 76* | 10.5* | 3 | 10 | 30.0 |
| el-Wad Terrace | ENAT | 23 | 332 | 6.9 | 18 | 41 | 43.9 |
| Hayonim Cave | ENAT | 38 | 99 | 38.4 | 15 | 54 | 27.8 |
| Hefzibah | GKEB | 3 | 514 | 0.6 | 111 | 508 | 21.9 |
| Neve David | GKEB | 4 | 47 | 8.5 | 23 | 67 | 34.3 |
| Nahal Hadera V | KEB | 79 | 1548 | 5.1 | 276 | 856 | 32.2 |
| Hayonim Cave | KEB | 3 | 70 | 4.3 | 26 | 69 | 37.7 |
| Meged Rockshelter | KEB | 1 | 28 | 3.6 | 10 | 38 | 26.3 |

*Data for P1 only.

P1=phalanx 1; P2=phalanx 2; MP=metapodial. UF=unfused.

Gazelle P1 and P2 fuse at 5-8 months of age. Gazelle MP fuses at 16-18 months of age (35, 36).
